# New insights on congenital pulmonary airways malformations revealed by proteomic analyses

**DOI:** 10.1186/s13023-019-1192-4

**Published:** 2019-11-28

**Authors:** C. Barazzone-Argiroffo, J. Lascano Maillard, I. Vidal, M. L. Bochaton-Piallat, S. Blaskovic, Y. Donati, B. E. Wildhaber, A.-L. Rougemont, C. Delacourt, I. Ruchonnet-Métrailler

**Affiliations:** 1 Pediatric Pulmonology Unit, Department of Pediatrics,Obstetrics and Gynecology, Children’s Hospital, 6 Rue Willy Donzé, 1211 Geneva, Switzerland; 2 Department of Pathology and Immunology, Faculty of Medicine, University of Geneva, Geneva, Switzerland; 3 Division of Pediatric Surgery, University Hospitals Geneva, University Center of Pediatric Surgery of Western Switzerland, Geneva, Switzerland; 4 Division of Clinical Pathology, Geneva University Hospitals, Geneva, Switzerland; 5 AP-HP, Hôpital Necker-Enfants Malades, Service de Pneumologie et Allergologie Pédiatriques, Paris, France; 6 Université Paris Descartes-Sorbonne Paris Cité, Paris, France

**Keywords:** Congenital pulmonary airways malformation, Lung malformations, Lung development, Proteomics

## Abstract

**Background:**

Congenital Pulmonary Airway Malformation (CPAM) has an estimated prevalence between 0.87 and 1.02/10,000 live births and little is know about their pathogenesis. To improve our knowledge on these rare malformations, we analyzed the cellular origin of the two most frequent CPAM, CPAM types 1 and 2, and compared these malformations with adjacent healthy lung and human fetal lungs.

**Methods:**

We prospectively enrolled 21 infants undergoing surgical resection for CPAM. Human fetal lung samples were collected after termination of pregnancy. Immunohistochemistry and proteomic analysis were performed on laser microdissected samples.

**Results:**

CPAM 1 and 2 express mostly bronchial markers, such as cytokeratin 17 (Krt17) or α-smooth muscle actin (ACTA 2). CPAM 1 also expresses alveolar type II epithelial cell markers (SPC). Proteomic analysis on microlaser dissected epithelium confirmed these results and showed distinct protein profiles, CPAM 1 being more heterogeneous and displaying some similarities with fetal bronchi.

**Conclusion:**

This study provides new insights in CPAM etiology, showing clear distinction between CPAM types 1 and 2, by immunohistochemistry and proteomics. This suggests that CPAM 1 and CPAM 2 might occur at different stages of lung branching. Finally, the comparison between fetal lung structures and CPAMs shows clearly different protein profiles, thereby arguing against a developmental arrest in a localized part of the lung.

## Introduction

Lung development is a complex process allowing parenchymal architecture to evolve along the bronchial organization. To establish correct bud elongation and airway branching, cellular interactions between epithelial, endothelial and mesenchymal cells are required. These interactions are dependent on the paracrine secretion of different growth factors or transcription factors. Growth factors are classified into different groups based on their cell of origin, such as fibroblast growth factors (FGF), vascular growth factors (VEGF), and epithelial growth factors (EGF). Transcription factors, such as SOX2 and SOX9, are recognized to play a role in lung development and in particular during branching morphogenesis [1–5]. During the canalicular stage, expression of SOX2 and SOX9 differ in their localization. Indeed, SOX 2 is expressed in the proximal airways surrounded with smooth muscle cells (SMCs) and SOX9 is restricted to the distal epithelial buds [1]. SMCs surrounding epithelial cells are crucial in this process due to their ability to contract and to allow SMCs later to extrude into branches [6, 7].

Congenital lung anomalies (CLA) are a group of developmental lung alterations thought to result from different external factors occurring during pregnancy, such as toxic exposure, or are associated to preterm birth. In these cases, cellular crosstalk can be altered or interrupted leading to the impairment of lung branching and alveolar formation [8–12]. Congenital pulmonary airway malformations (CPAM) belong to a group of rare CLA whose pathological origin is still poorly understood [13]. In Western Europe, CPAM have an estimated prevalence between 0.87 and 1.02/10,000 live births [14]. Depending on timing of routine ultrasound, CPAM are often detected around 16 to 20 gestational weeks (GW). CPAM were initially classified by Stocker et al. in 3 different subtypes of cystic lung lesions (1 to 3), differing both macroscopically (cyst size) and on histology [13]. Despite further attempts at refining the categories, a type 0, or congenital acinar dysplasia and a type 4 category, representing pneumopulmonary blastoma instead of CPAM were added [15]. Langston preferred the denomination “large cyst and small cyst-types”, i.e. type 1 and 2, the definition used in this paper [16]. It remains as yet unclear whether or not CPAM 1 and 2 share the same origin.

Based on these considerations, the current research project aims at studying by several approaches the cellular origins of the two most frequent CPAM, CPAM types 1 and 2 (0.85/10,000 and 0.2/10,000 live births, respectively). We stained surgically removed CPAM specimens and analyzed markers of alveolar, muscular and bronchial cell differentiation on these samples. Adjacent healthy lung parenchyma served as control. We found that cystic epithelium from both CPAM subtypes expresses several bronchial markers. On the other hand, SPC, a marker of alveolar epithelial type 2 cells (AECII), was expressed in CPAM 1, but barely seen in CPAM 2. We then assessed ACTA2 expression and its distribution in CPAM. Here again, we observed similarities in terms of ACTA2 expression in SMCs of both CPAM 1 and bronchi, whereas ACTA2 positive SMCs were less prevalent in CPAM 2. These results were further reinforced by proteomic analysis performed on CPAM cysts, and healthy adjacent normal-appearing lung, as well as on fetal airspace and bronchial epithelium, after microlaser dissection. CPAM 2 protein profile was clearly distinct from all the other samples. Furthermore, CPAM protein profiles overlapped partially with those of fetal samples. Our data provide important insights into CPAM origin and demonstrate some differences between CPAM types 1 and 2, suggesting that these malformations might occur at different stages of embryogenesis.

## Materials and methods

### Study design, subjects and description of types of lesions

Twenty-one children with CPAM diagnosed by antenatal ultrasound were prospectively enrolled at the Children’s hospital of Geneva at time of surgery from November 2012 to November 2017. The institutional ethics committee approved this study and informed consent was obtained during scheduled hospital visits (CER 12–110). Classification of the CPAM types was established by the pathologist upon macroscopic examination of the specimens (Additional file 4). Analyses were performed on CPAM tissue and on healthy non-cystic adjacent lung, considered as control lung. Human fetal samples were collected after termination of pregnancy. The age of the fetuses ranged from 14 to 16 weeks of gestation. The institutional ethics committee approved this procurement and informed consent was obtained from the parents (PB_2016–00175).

### Immunohistochemistry and image acquisition

Five μm slides were cut from formalin-fixed paraffin-embedded (FFPE) tissue blocs for immunohistochemistry (IHC). Samples of CPAM 1, CPAM 2 and control lung as well as human fetal samples were assayed on two separate experiments. High-resolution pictures of immunostained slides were acquired using a brightfield slide scanner microscope (Axioscan Z.1, ZEISS), using a 10x magnification. Antibodies used and quantification methods are detailed in the Additional file 4.

### Protein extraction, laser microdissection (LMD) and mass spectrometry analysis

For total lung extracts, 34 samples (14 CPAM 1, 7 CPAM 2, 13 control lung) were analyzed across 4 different experiments (see Additional file 4). Protein extraction was performed using the mild anionic detergent RapiGest - SF (Waters Corporation, Massachussets, USA). For the Laser microdissection experiment, 16 samples (4 CPAM 1 cyst borders, 3 CPAM 2, 3 control bronchi, 3 control alveolar areas, 3 fetal canaliculi and 3 fetal bronchi) were analyzed across two different experiments. Proteins were reduced, alkylated and digested with trypsin. Resulting peptides were sequenced by liquid chromatography coupled with tandem mass spectrometry (LC-MS/MS) at the Proteomic core facility of the faculty of medicine of the University of Geneva. The resulting characteristic peptide fragmentation spectra were then blasted to the SWISSPROT protein sequence database. Database search was performed with Mascot Server (Matrix Science Ltd., London, UK) and results were analyzed and validated using Scaffold software (Proteome software Inc., Oregon, USA).

### Imaging and statistics

Analysis of staining was blinded and independently performed by two of the authors. Ten random epithelial zones of 3 to 7 different patients/conditions were analyzed at magnification × 10. Staining quantification was performed using image J software [17] and a mean ± SD was calculated for each patient (see Additional file 4). The different means from each patient were then added to calculate a SEM. Data are presented as average values ± SEM. Statistical analysis was performed using GraphPad Prism software (GraphPad Software, California, USA). One-way ANOVA were used to compare groups. The results were considered significant if *p* < 0.05.

### Proteomic data analysis

For each sample, the number of peptides assigned to each protein was normalized to the total number of peptides obtained in the same sample. Unsupervised hierarchical clustering was computed using the R language and environment (v 3.5.3) (https://www.r-project.org), and the “pheatmap” package (v1.0.12) [18]. Functional annotation was performed using the R packages “AnnotationDbi” (v1.44.0), “org. Hs.eg.db” (v 3.7.0) and “GO.db” (v 3.7.0).

## Results

### Patient characteristics

A total of 21 patients were included: 14 (56%) CPAM 1 and 7 (28%) CPAM 2. Four CPAM 1 and 5 CPAM 2 were associated with intralobar bronchial sequestration. Most of patients were born at term (median age 39 weeks of gestation) with a birth weight adapted to the gestational age (median weight 3290 g) (Table 1). Four patients presented respiratory failure attributable to the lung lesion and needed mechanical ventilation at birth. In 3 patients, CPAM were associated with other malformations. CPAM classification was determined by the pathologist prior to IHC and proteomic analysis.

**Table 1:** Patient characteristics

Lesion Type	CPAM 1	CPAM 2
Number	14	7
Birthweight (1)	3400 [1650–3770]	3290 [1910–4000]
Male sex	5	4
GA at birth (2)	39 [31–40]	40 [36–41]
Antenatal diagnostic age (3)	20 [17–27]	23 [21–27]
Need for mechanical ventilation (4)	3	1
Associated sequestrations	4	5
Other malformations/ syndromes	*n* = 2 (lung hypoplasia, vertebral anomaly)	*n* = 1 (palatine cleft, cardiac malformation, cerebral malformation)

1. Birthweight reported in grams – median [range]

2. Gestational age (GA) reported in weeks – median [range]

3. Age reported in weeks of gestation- median [range]

4. Patients with a medical indication other than the lung malformation were excluded from this column

### Epithelial pulmonary cells express SOX2 and SOX9 during fetal development and in cystic lung

**Fig. 1:**
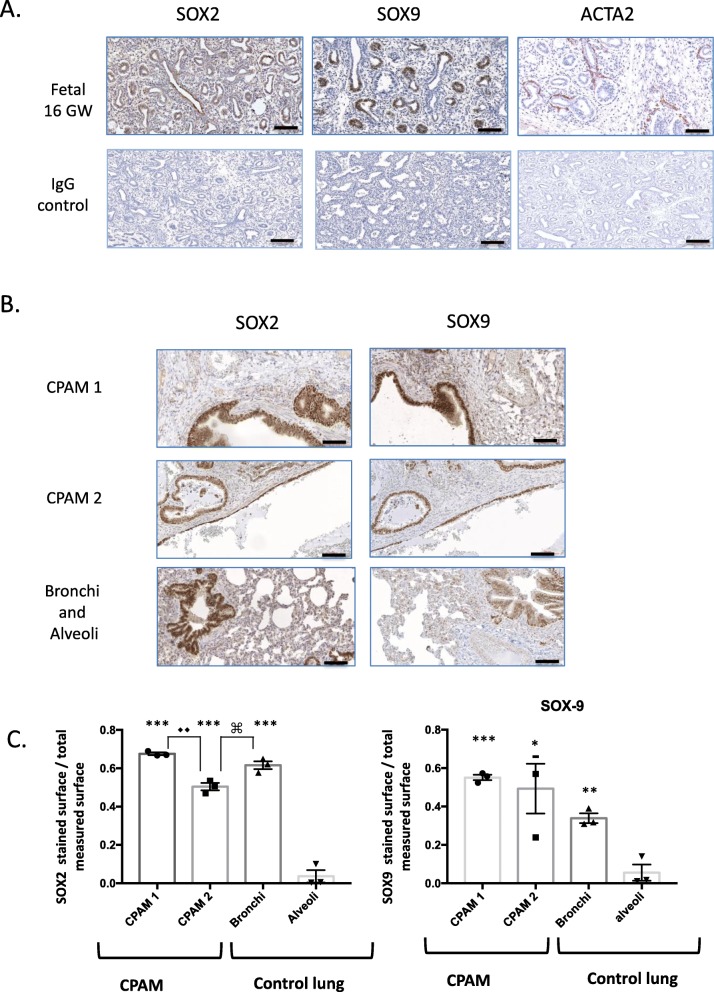
Expression of SOX2 and SOX9 during fetal development, and in CPAM cysts and control lung. **a**. Immunohistochemical staining of human fetal lung tissue at 16 weeks of gestation (canalicular stage) with SOX2, SOX9 and ACTA2 antibodies. Note the differential expression of SOX2 and SOX9 at the tip of the buds. **b**. Immunostaining of CPAM 1, CPAM 2 and control lung with SOX2 and SOX9 antibodies. Note the strong staining of the bronchial epithelium and of the epithelial cyst lining **c**. Graph comparing SOX2 and SOX9 stained surface with the total measured surface in CPAM and in control tissue. Alveoli compared to all other samples: *** *p* < 0.001, ** *p* < 0.01. * *p* < 0.05. CPAM 1 compared to CPAM 2 ♦♦ *p* < 0.01, CPAM 2 compared to Bronchi ⌘ *p* < 0.05. *N* = 3 different patients, each point corresponds to the mean of 10 random pictures per patient. Scale bars: 100 μm

We first analyzed the expression of the two transcription factors, SOX2 and SOX9 in fetal lung at 16 GW obtained from human fetuses. Indeed, lung branching depends on the proximal to distal airways gradient of these different transcription factors that influence epithelial progenitors [3]. SOX2 was present not only at the tips, but also partially around the growing buds, similarly to ACTA 2 a marker of SMCs (Fig. 1a). SOX9 was principally localized at the tips of elongating buds. We then looked at the expression of SOX2 and SOX9 in CPAM samples. Both transcription factors were diffusely expressed in the epithelial cells lining the cysts (Fig. 1b). SOX2 was significantly less expressed in CPAM 2 compared to CPAM 1. In the control lung, SOX2 and SOX9 were also diffusely present in bronchial epithelial cells and significantly less in alveoli as attested by quantification (Fig. 1c).

### Smooth muscle cells are present in high amount around the cysts

**Fig. 2:**
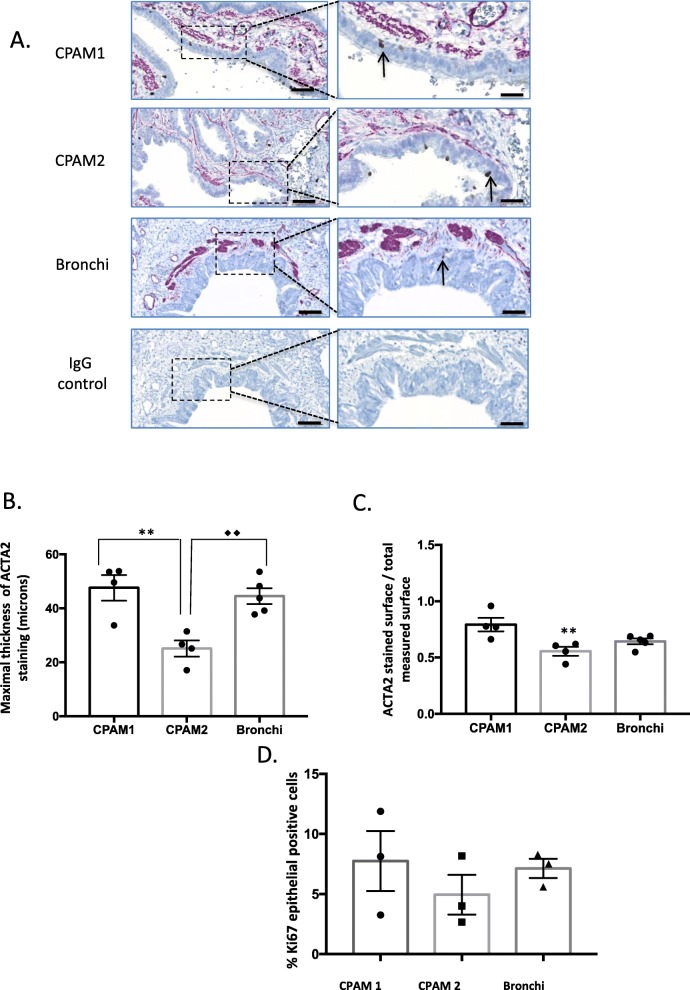
Smooth muscle cell (SMC) distribution and Ki67 expression in CPAM and in control lung tissue. Immunostaining of CPAM 1, CPAM 2 and control lung with ACTA2 and Ki67 antibodies. **a**. CPAM 1, CPAM 2 and adjacent control lung sections co-stained with anti-ACTA2 (purple) and anti-Ki67 (dark brown, black arrows in the magnified area at the right panel). **b**. Comparison of the maximal thicknesses of ACTA2 staining in bronchi, CPAM 1 and CPAM 2 in microns. CPAM 1 compared to CPAM 2 sample: ** *p* < 0.01. Bronchi compared to CPAM 2 sample: ♦♦ p < 0.01. **c**. Graph comparing the ratio of ACTA2 stained surface to the total measured surface. CPAM 1 compared to CPAM 2: ** *p* < 0.05. *N* = 3 different patients, each point corresponds to the mean of 10 random pictures per sample. **d**. Graph comparing the percentages of Ki67 epithelial positive cells between groups. *N* = 3 different patients, each point corresponds to the mean of 10 random pictures per sample. Scale bars: 100 μm

SMCs are described to exert a central role in driving branching morphogenesis, possibly due to their ability to contract and induce airway peristalsis [16, 19]. In the adult lung, various layers of smooth muscle surround the bronchi in a spiral conformation and their thickness decreases from proximal to distal airways [20]. We observed a different distribution of ACTA2-positive cells between CPAM 1 and CPAM 2, as shown in Fig. 2a. Immunoreactivity to ACTA2 was used to determine SMC thickness in the CPAM cyst walls. In CPAM 2, SMC thickness assessed by ACTA2 was significantly lower than in CPAM 1 and in bronchi (*p* < 0.05) (Fig. 2 b). We then analyzed the ACTA2 positive area fraction in each sample. ACTA2 positive surface was larger in CPAM 1 than in CPAM 2 (p < 0.05) (Fig. 2 c). Cell distribution around the cysts was also different: CPAM 1 cysts presented a more continuous SMC layer compared to discontinuous ACTA2 SMC layers surrounding bronchi in CPAM 2. In order to determine whether the presence of ACTA2 coincides with epithelial proliferation, we co-stained our samples with ACTA2 and Ki67 (Fig. 2 a, d). We did not detect any differences in cell proliferation related to ACTA2 thickness. However at this point, we cannot exclude a sequential phenomenon, with epithelial cells first proliferating, followed by an increase in SMC layers.

### Congenital lung cysts are lined by differentiated respiratory epithelium

**Fig. 3:**
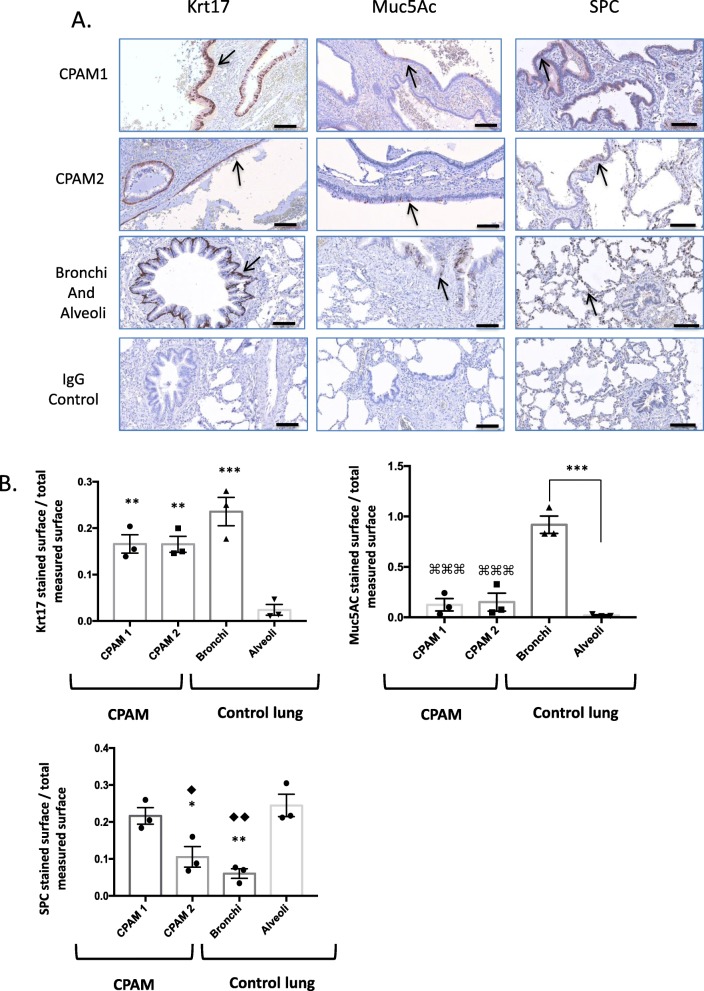
Epithelium from lung cysts express markers of differentiated airways. **a**. Immunostaining of CPAM 1, CPAM 2 and control lungs with Krt17, Muc5AC and SPC antibodies (black arrows). Scale bars: 100 μm **b**. Graph comparing the ratio of stained surfaces to the total measured surfaces for Krt17, Muc5AC and SPC staining in CPAM and control tissue. Alveoli compared to other samples: ** *p* < 0.01, *** *p* < 0.001, Bronchi compared to other samples: ⌘⌘⌘ p < 0.001, CPAM 1 compared to CPAM 2: ♦ *p* < 0.05, CPAM 1 compared to bronchi: ♦♦ *p* < 0.01. N = 3 different patients, each point corresponds to the mean of 10 random pictures per patient

We then compared, by IHC staining, the cellular types surrounding the cystic epithelium and adjacent lung for different specific markers of differentiated airway structures (Fig. 3 a). Bronchial epithelial cells express Krt17 [21]. Similarly, epithelial cells surrounding the cysts expressed this marker. Muc5Ac, a specific marker of goblet cells, was highly expressed in bronchi and significantly less so in alveoli and CPAM samples (*p* < 0.001) (Fig. 3b). Accordingly, mucinous cells were not observed in the CPAM samples submitted to analysis (data not shown).

As expected, we detected strong staining for SPC, a specific marker of AEC II, in the alveoli of control adjacent lung, and only weak staining in bronchi. SPC positive cells in CPAM 2 were significantly less than in alveoli (*p* < 0.05). Unexpectedly, CPAM 1 cysts expressed appreciable amounts of SPC without significant differences with normal-appearing alveoli. All together, these results suggest that the cystic epithelium of CPAM 1 and 2 expresses markers of differentiated bronchial epithelium, but only CPAM 1 seems to express significantly higher amounts of SPC, an alveolar cell marker.

### Proteomic profiles of congenital lung lesions

We then analyzed the different protein profiles of CPAM 1 and 2, after laser microdissection (LMD). We first attempted to identify protein profiles on total lung samples either from CPAM or control lung, but failed to find any differences between the groups. We then focused on the epithelium lining the CPAM cysts, and on bronchial epithelium of control lung (Additional file 1: Fig. S1 A). We also looked at the airspaces (canaliculi) and bronchi of fetal tissue.

**Fig. 4:**
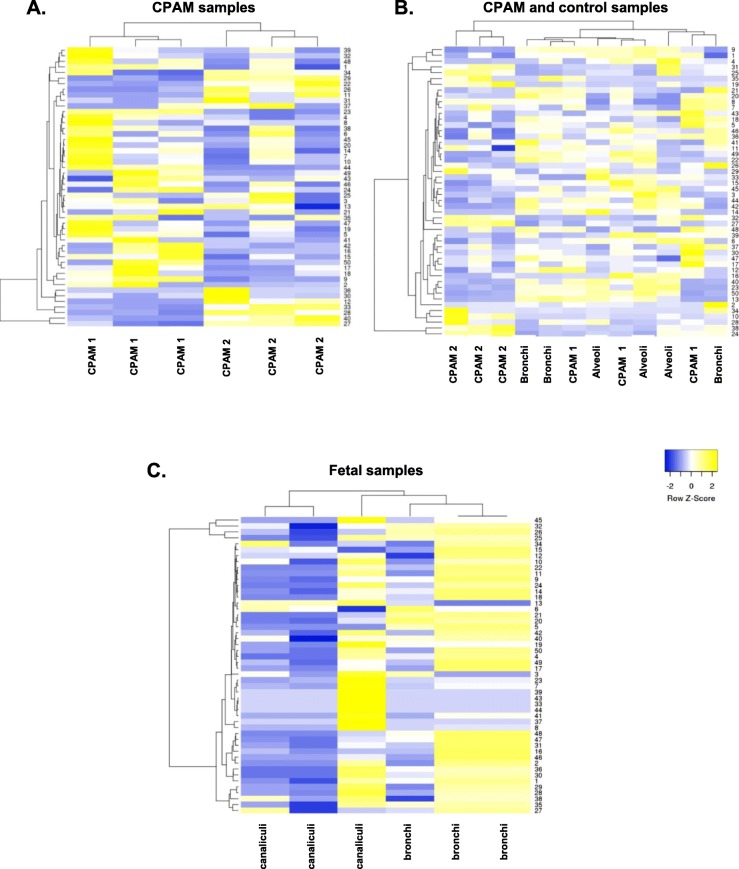

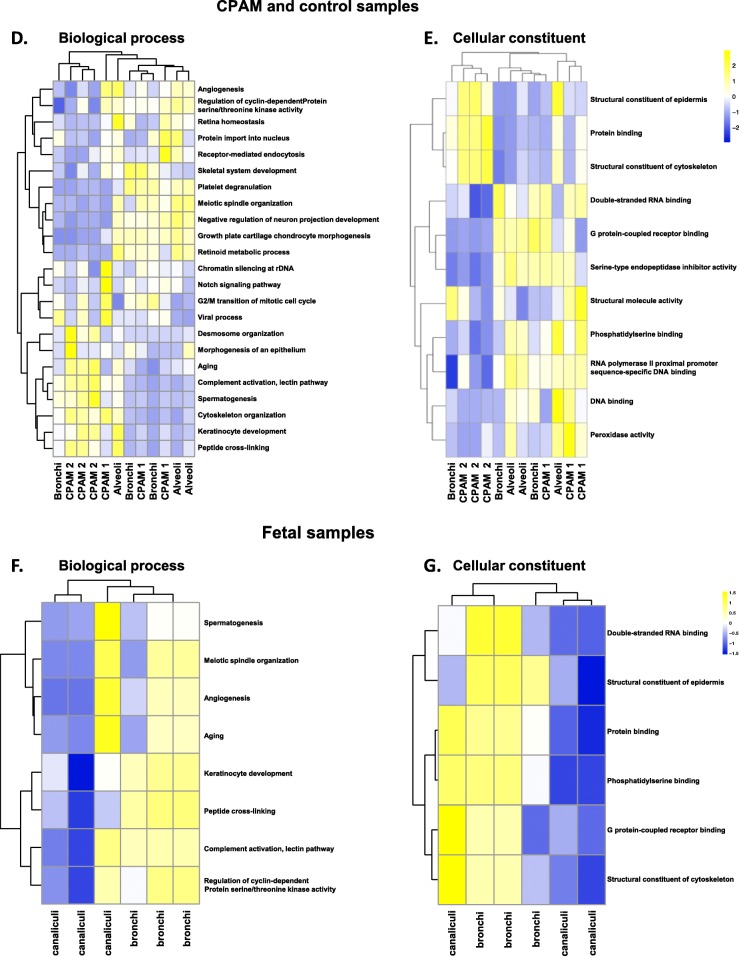
Protein profiles of CPAM cyst epithelium, bronchi, alveoli and fetal tissue. Proteins were obtained by microlaser dissection from 3 different patients. **a**-**c** Hierarchical clustering was based on the top 50 more abundant proteins. **a**. Comparison of proteomic profiles from the epithelial lining of CPAM 1 and CPAM 2 cysts, obtained by laser microdissection. Of note, CPAM 1 and CPAM 2 form two distinct clusters. **b**. Comparison of proteomic profiles from CPAM 1 or CPAM 2 cyst epithelium, and control lung tissues (bronchi and alveoli). CPAM 2 cluster together and CPAM 1 samples cluster close to bronchi and are more distant from the alveoli. **c**. Comparison of proteomic profiles from epithelial lining of fetal canaliculi and bronchi (16 GW). **d**-**e** Hierarchical clustering was based on the entire set of proteins detected. **d**. Protein-set enrichment analysis of biological process from the epithelial lining of CPAM 1 and CPAM 2 cysts, obtained by laser microdissection. **e**. Protein-set enrichment analysis of cellular constituent from the epithelial lining of CPAM 1 and CPAM 2 cysts, obtained by laser microdissection. **f**-**g**. Hierarchical clustering of fetal samples was based on a selection of candidate GO terms. **f**. Selected protein-set enrichment analysis of biological process from the epithelial lining from fetal canaliculi and bronchi (16 GW). **g**. Selected protein-set enrichment analysis of cellular constituent from the epithelial lining from fetal canaliculi and bronchi (16 GW).

Side by side analysis of protein profiles of microdissected epithelium from 3 different patients of each groups (CPAM 1, CPAM 2 and control lungs) was performed using Euclidean distance and the complete linkage method for clustering. Data were normalized using the following formula ($normalized\ peptide\ number=\frac{number\ of\ peptide s\  per\  protein}{number\ of\ total\ peptides\  per\  sample}$). Hierarchical clustering based on the expression levels of the top 50 identified proteins (Additional file 2: Figure S2 A-C) showed clear separate clusters between CPAM 1 and CPAM 2 (Fig. 4a). CPAM 1 cluster was less homogenous than that of CPAM 2. Indeed, CPAM 1 cystic epithelium clustered closer to bronchial and alveolar samples than CPAM 2, which formed a distinct cluster (Fig. 4b). These findings confirm our previous IHC results in that CPAM 1, although sharing several markers with bronchi, also expresses alveolar epithelial cell markers (SPC). We then compared the fetal canaliculi and bronchus protein profiles. Separate clusters were obtained between the two groups, with the exception of one fetal canaliculi sample (Fig. 4c).

CPAM 1 upregulated proteins were analyzed in CPAM 2, fetal bronchi and fetal canaliculi. A short list of 5 upregulated proteins in CPAM 1 upregulated proteins was found to be similarly upregulated in fetal bronchi, as compared to CPAM 2 and fetal canaliculi (Additional file 3: Figure S3C). This result suggests that some degree of similarity exists between fetal bronchi and CPAM 1 (Additional file 3: Figure S3A). These proteins were assigned by GO term to epithelial/epidermal differentiation, and to the organization of adherens junctions and intermediate filaments (GO consortium and DAVID bioinformatics tools) with statistical significance (FDR < 0.05).

In addition, proteins were annotated into biological process and cellular constituent with again distinct clusters found between CPAM 1 and CPAM 2 (Fig. 4d-e).

CPAM 2 samples showed an enrichment of proteins linked to the cytoskeleton organization, aging, spermatogenesis and keratinocyte development and a decrease in proteins involved in the regulation of cyclin-dependent protein kinase activity and angiogenesis, compared to CPAM 1 or control lung tissue (bronchi and alveoli).

We then analyzed, in fetal samples, a subgroup of the GO terms found in control lung tissue and CPAM samples (Fig. 4 f-g). We found that most of the biological process and cellular constituent were higher expressed in bronchi than in canaliculi.

Both CPAM 2 and most of fetal canaliculi exhibit a downregulation of cell cycle regulator or extracellular matrix component (Fig. 4 d and f).

## Discussion

In this study, we used two different approaches, IHC and proteomics, to characterize CPAM lesions. Several studies have been dedicated to the analysis of the growth and transcription factors implicated in the cellular proliferation of altered lungs [8]. The results have highlighted the role of different molecules, such as FGF-7, FGF-10, PDGF BB and HOXB5, in abnormal lung development [22, 23]. All these studies were performed in animal models mimicking CPAM, but only few experiments have been realized to date on human samples [24–29]. In humans, the characterization and classification of the different types of CPAM is based on histopathological evaluation, as a first guide for assessing phenotypic variation and subtyping. CPAM types 1 and 2 differ in both cyst size and histology (cell types lining the cysts, muscle wall, presence or absence of cartilage among others) [13, 15, 16]. CPAM are therefore mainly classified according to gross findings, histological features and the structures along the respiratory tract they most resemble. However these descriptions do not integrate embryological pathogenesis.

To provide better insight in possible links with the embryological development of the lung, we analyzed the expression of SOX2 and SOX9 in growing buds during lung canalicular developmental stage and also observed an antero-posterior gradient in SOX expression, as previously reported [1]. This gradient was not present in control postnatal bronchi, neither in CPAM cysts. Indeed, SOX2 and SOX9 staining was similar along CPAM epithelium and adjacent bronchi. Notwithstanding, CPAM 2 lesions expressed significantly less SOX2 than CPAM 1.

SOX2 positive cells are tightly regulated by the presence of ACTA2 positive cells that allow for branching in parallel to a decrease in SOX2/ SOX9 positive cells during the canalicular stage [1]. Danopoulos and colleagues suggested an interaction between SOX2/ SOX9 cells and SMCs that could possibly influence cell proliferation in the growing airways of the human fetal lung [1]. Indeed, SMCs are essential to regulate epithelial branching through peristalsis, paracrine-signalling pathways and secondary lung septation [7]. In CPAM, although we observed significant differences in SMC distribution, with CPAM 1 being closer to bronchi, we could not find a correlation between the SMC thickness and epithelial cell proliferation.

Specific epithelial cell marker analysis showed some similarities between bronchi and CPAM epithelium, suggesting abnormal epithelial proximo-distal differentiation. Interestingly, only the CPAM 1 epithelium contained SPC positive cells, a specific staining for late progenitors and differentiated AECII. The increased number of SPC positive cells can be due to the differentiation process. Indeed, bronchioalveolar stem cells express SPC and give later rise to AECII. Alternatively, the presence of pro-SPC in these cysts might suggest that the initial event happens later during embryogenesis stage when alveolar cells are already differentiated.

In contrast to the study by Swarr et al., no mucinous cell clusters were seen in the analyzed CPAM samples [30]. Furthermore, MUC5AC expression in CPAM 1 and CPAM 2 was significantly lower than in bronchi (Fig. 3b). This result suggests that although CPAM epithelium might derive from bronchial tree, significant differences are observed between CPAM and bronchial epithelium.

After microlaser dissection of the different epithelial linings, we detected distinct protein clusters in CPAM 2 and CPAM 1 corresponding to the preliminary pathologist classification. Indeed, CPAM 2 presented a distinctive proteomic profile compared to CPAM 1, bronchi and alveoli. CPAM 1 clustered with alveoli and bronchi, thereby corroborating our immunohistochemistry results, where CPAM 1 showed some similarities with bronchi concerning ACTA2, Krt17 and SOX2/9 staining, but also with alveoli.

These results call for two different hypotheses: either CPAM originates from the developing bronchi at different stages of development, or these lesions represent truly distinct entities resulting from a different etiology. The similarities between CPAM 1 and both bronchi and alveoli, and of CPAM 2 only with bronchi reinforce the hypothesis that CPAM 1 and CPAM 2 grow at different lung branching timelines. A subset of proteins is upregulated in CPAM 1 and fetal bronchi, as compared to CPAM 2 and to fetal canaliculi. This suggests a similarity between CPAM 1 and fetal bronchi. Contrariwise, no clear link was observed between CPAM 2 and fetal proteins. The comparison between fetal canaliculi, bronchi and CPAM identified clearly distinct protein profiles between fetal tissue and CPAM 2, whereas in CPAM 1 some fetal bronchial proteins remained detectable.

A modest reduction of PI3K-AKT-mTOR signalling pathway was suggested to influence CPAM 1 and CPAM 2 formation in transcriptomic data [30]. We also found in our proteomic analyses a down regulation of phosphatidylserine binding protein, an AKT activation modulator, supporting this finding (Fig. 4e) [31]. The same result was observed in most of fetal canaliculi (Fig. 4g). Similarly to Swarr et al., we report that CPAM 2 upregulated proteins were involved in cellular proliferation and differentiation (cytoskeleton organization, spermatogenesis and keratinocyte development). In addition, filament and microtubule organization are important to allow correct cellular arrangement. Upregulation of these biological process confirmed previous published transcriptomic data [30].

Finally, the heterogeneity in the CPAM 1 cluster compared to bronchi and alveoli could suggest the existence of intermediate phenotypes reinforcing the overlapping features often seen histologically. The upregulated proteins seen in both CPAM 1 and fetal bronchi, but not in CPAM 2 and fetal canaliculi have a role in mesenchyme-epithelial differentiation or cytoskeletal formation. These proteins have been involved in tumorigenesis due to their role in proliferation and differentiation pathways, as well as in cellular crosstalk during lung embryogenesis [32–34]. This last result links CPAM and alterations in cellular crosstalks with abnormal desmosome communications that could suggest a different physiopathological etiology in CPAM 2. The link with tumorigenesis is unclear, since malignancy in CPAM is rather related to the presence of clusters of mucinous cells, seen in CPAM 1 but not in CPAM 2 Higher numbers of CPAM 2 samples should be analyzed to confirm these results.

Our results are thus in agreement with the transcriptomic analyses already published showing a distinction between cyst and control lung [30]. Nevertheless our experiments add a more precise distinction between the epithelium present in cysts and control tissue samples due to the microlaser dissected epithelium analysis.

Our study has some limitations. First, although our findings were confirmed by proteomic analysis, sample size is small and need to be enlarged. Second, the prevalence of CPAM 1 and CPAM 2 variants can differ depending of the center of recruitment with more cases of mucinous cell clusters leading to potential tumoral transformation [35, 36]. We observed the presence of different keratins in our proteomic results. Finally, according to different animal studies, SMCs may influence CPAM formation due to the secretion of growth factors [27, 28]. Unfortunately, even if we suspect a role of the mesenchyme in CPAM formation, proteomic analysis was not able to identify in this study the previously involved growth factors, and only a minority of the transcriptional factors described in lung malformations in animal models. Protein crosslink due to FFPE conservation could have influenced our results, by allowing for only partial protein detection, the most resistant being mainly structural proteins. However, our results are in accordance with the previous transcriptomic study by Swarr et al., who find differences between CPAM malformation types [30].

## Conclusion

The results reported in our study provide a new step in the understanding of CPAM etiology. This study is the first on CPAM to our knowledge, to use a proteomic approach with lung samples obtained after microlaser dissection. This exciting method allows for the analysis of different compartments within the CPAM lesions. This methodology applied to CPAM lesion is innovative and the possible use of FFPE material will allow for the analysis of tissue samples from different biobanks, avoiding the shortage of frozen material. Interestingly, proteomic differences observed between CPAM 1 and 2 support to the initial pathological classification proposed by Stocker et al., and by the revision provided by Langston more than the recent classification using micro and macrocysts classification [30, 37]. This technique could also help in the diagnosis of CPAM subtypes in unclear clinical cases.

Future work including more patients and quantitative proteomic analyses could pave the way to a more in-depth delineation between CPAM types 1 and 2. In conclusion, the description and classification of CPAM lesions remains a real challenge, the main issues being adequate management decisions for these patients.

## Supplementary information

**Additional file 1: Figure S1.** Illustration of the recovered samples obtained by laser microsdissection. A. Selected areas B. Area cut with laser microdissection (Dissected area, lower panels). Red scale bars: 100 μm.

**Additional file 2: Figure S2.** List of the 50 recovered proteins. A. List of proteins present in CPAM 1 and CPAM 2 B List of proteins present in CPAM 1, CPAM 2 and control tissue C. List of proteins present in fetal canaliculi and bronchi at 16 GW.

**Additional file 3: Figure S3.** List of protein candidates. A. List of proteins significantly upregulated in CPAM 1 and fetal bronchi, as compared to CPAM 2 and fetal canaliculi determined with the GO tool from PANTHER (GO consortium, PANTHER, maintained by Thomas laboratory, University of Southern California). B. List of proteins significantly upregulated in CPAM 2 and the possible pathways in which they are involved, determined with the GO tool from PANTHER (GO consortium, PANTHER, maintained by Thomas laboratory, University of Southern California).

**Additional file 4.** Supplementary material and methods.

## Data Availability

Please contact author for data requests

## Abbreviations

ACTA 2: α-smooth muscle actin
AEC II: alveolar epithelial type 2 cells
CLA: congenital lung anomalies
CPAM: congenital pulmonary airways Malformation
FFPE: formalin-fixed paraffin-embedded
GW: gestational weeks
IHC: immunohistochemistry
Krt17: cytokeratin 17
SMCs: smooth muscle cells
SOX: SRY-box
SPC: surfactant protein C
