# Supplementary figures and images for: New insights on congenital pulmonary airways malformations revealed by proteomic analyses
Source: Orphanet J Rare Dis. 2019 Nov 28;14:272. doi: 10.1186/s13023-019-1192-4 (PMC6883702; doi:10.1186/s13023-019-1192-4)

Figure S1

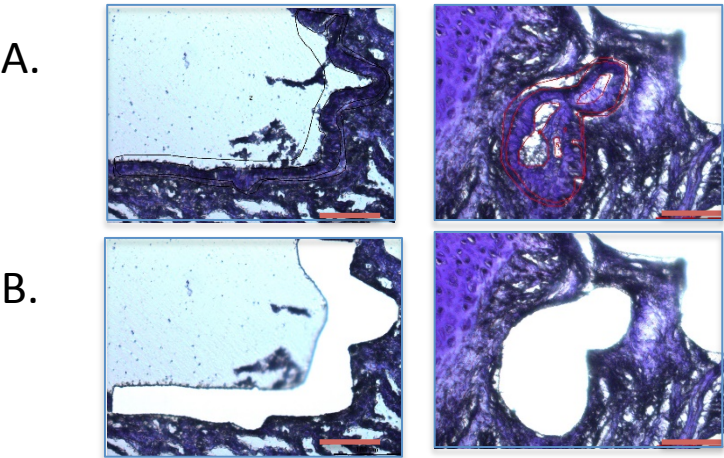

Supplement: Supplementary file 1 — Additional file 1: Figure S1. Illustration of the recovered samples obtained by laser microsdissection. A. Selected areas B. Area cut with laser microdissection (Dissected area, lower panels). Red scale bars: 100 μm. [file 13023_2019_1192_MOESM1_ESM.pdf]
