# Supplementary material for: New insights on congenital pulmonary airways malformations revealed by proteomic analyses
Source: Orphanet J Rare Dis. 2019 Nov 28;14:272. doi: 10.1186/s13023-019-1192-4 (PMC6883702; doi:10.1186/s13023-019-1192-4)
Supplement: Supplementary file 2 — Additional file 2: Figure S2. List of the 50 recovered proteins. A. List of proteins present in CPAM 1 and CPAM 2 B List of proteins present in CPAM 1, CPAM 2 and control tissue C. List of proteins present in fetal canaliculi and bronchi at 16 GW. [file 13023_2019_1192_MOESM2_ESM.pdf]

Figure S2

A.

	Accession		Protein name	Gene Name	Sequence length (a.a.)
1	P60709	ACTB_HUMAN	Actin, cytoplasmic 1 (Beta-actin)	ACTB	375
2	P02768	ALBU_HUMAN	Serum albumin	ALB	609
3	P00352	AL1A1_HUMAN	Retinal dehydrogenase 1 (RALDH 1)	ALDH1A1	501
4	P04083	ANXA1_HUMAN	Annexin A1 (Annexin I)	ANXA1	346
5	P07355	ANXA2_HUMAN	Annexin A2 (Annexin II)	ANXA2	339
6	P09525	ANXA4_HUMAN	Annexin A4 (35-beta calcimedin)	ANXA4	319
7	P25705	ATPA_HUMAN	ATP synthase subunit alpha, mitochondrial	ATP5F1A	553
8	Q13938	CAYP1_HUMAN	Calcyphosin (Calcyphosine)	CAPS	275
9	P12111	CO6A3_HUMAN	Collagen alpha-3(VI) chain	COL6A3	3177
10	H7BZ55	CRCC2_HUMAN	Putative ciliary rootlet coiled-coil protein 2	CROCC2	1655
11	Q02413	DSG1_HUMAN	Desmoglein-1 (Cadherin family member 4)	DSG1	1049
12	P15924	DESP_HUMAN	Desmoplakin (DP)	DSP	2871
13	P68104	EF1A1_HUMAN	Elongation factor 1-alpha 1 (EF-1-alpha-1)	EEF1A1	462
14	P15311	EZRI_HUMAN	Ezrin (Cytovillin) (Villin-2)	EZR	586
15	P35555	FBN1_HUMAN	Fibrillin-1	FBN1	2871
16	P21333	FLNA_HUMAN	Filamin-A (FLN-A)	FLNA	2647
17	P69905	HBA_HUMAN	Hemoglobin subunit alpha (Alpha-globin)	HBA1	142
18	P68871	HBB_HUMAN	Hemoglobin subunit beta (Beta-globin)	HBB	147
19	P62805	H4_HUMAN	Histone H4	HIST1H4A	103
20	P22626	ROA2_HUMAN	Heterogeneous nuclear ribonucleoproteins A2/B1 (hnRNP A2/B1)	HNRNPA2B1	353
21	P61978	HNRPK_HUMAN	Heterogeneous nuclear ribonucleoprotein K (hnRNP K)	HNRNPK	463
22	Q86YZ3	HORN_HUMAN	Hornerin	HRNR	2850
23	P07900	HS90A_HUMAN	Heat shock protein HSP 90-alpha (Heat shock 86 kDa)	HSP90AA1	732
24	P0DMV8	HS71A_HUMAN	Heat shock 70 kDa protein 1A (Heat shock 70 kDa protein 1)	HSPA1A	641
25	P04792	HSPB1_HUMAN	Heat shock protein beta-1 (HspB1)	HSPB1	205
26	P14923	PLAK_HUMAN	Junction plakoglobin (Catenin gamma) (Desmoplakin III)	JUP	745
27	P04264	K2C1_HUMAN	Keratin, type II cytoskeletal 1	KRT1	644
28	P13645	K1C10_HUMAN	Keratin, type I cytoskeletal 10 (Cytokeratin-10)	KRT10	584
29	P02533	K1C14_HUMAN	Keratin, type I cytoskeletal 14 (Cytokeratin-14) (CK-14) (Keratin-14) (K14)	KRT14	472
30	P08779	K1C16_HUMAN	Keratin, type I cytoskeletal 16 (Cytokeratin-16)	KRT16	473
31	Q04695	K1C17_HUMAN	Keratin, type I cytoskeletal 17 (39.1) (Cytokeratin-17)	KRT17	432
32	P08727	K1C19_HUMAN	Keratin, type I cytoskeletal 19 (Cytokeratin-19)	KRT19	400
33	P35908	K22E_HUMAN	Keratin, type II cytoskeletal 2 epidermal (Cytokeratin-2e)	KRT2	639
34	P13647	K2C5_HUMAN	Keratin, type II cytoskeletal 5 (58 kDa cytokeratin) (Cytokeratin-5)	KRT5	590
35	P02538	K2C6A_HUMAN	Keratin, type II cytoskeletal 6A (Cytokeratin-6A)	KRT6A	564
36	P04259	K2C6B_HUMAN	Keratin, type II cytoskeletal 6B (Cytokeratin-6B)	KRT6B	564
37	P48668	K2C6C_HUMAN	Keratin, type II cytoskeletal 6C (Cytokeratin-6C)	KRT6C	564
38	P08729	K2C7_HUMAN	Keratin, type II cytoskeletal 7 (Cytokeratin-7)	KRT7	469
39	P05787	K2C8_HUMAN	Keratin, type II cytoskeletal 8 (Cytokeratin-8)	KRT8	483
40	P35527	K1C9_HUMAN	Keratin, type I cytoskeletal 9 (Cytokeratin-9)	KRT9	623
41	P02545	LMNA_HUMAN	Prelamin-A/C [Cleaved into: Lamin-A/C (70 kDa lamin)	LMNA	664
42	P35579	MYH9_HUMAN	Myosin-9 (Cellular myosin heavy chain, type A) (NMMHC-A)	MYH9	1960
43	P14618	KPYM_HUMAN	Pyruvate kinase PKM (EC 2.7.1.40) (Cytosolic thyroid hormone-binding protein)	PKM	531
44	Q15149	PLEC_HUMAN	Plectin (PCN) (PLTN)	PLEC	4684
45	Q06830	PRDX1_HUMAN	Peroxiredoxin-1 (EC 1.11.1.15) (Natural killer cell-enhancing factor A)	PRDX1	199
46	P60174	TPIS_HUMAN	Triosephosphate isomerase (TIM)	TPI1	286
47	Q71U36	TBA1A_HUMAN	Tubulin alpha-1A chain (Alpha-tubulin 3) (Tubulin B-alpha-1)	TUBA1A	451
48	P68371	TBB4B_HUMAN	Tubulin beta-4B chain (Tubulin beta-2 chain)	TUBB4B	445
49	P0CG47	UBB_HUMAN	Polyubiquitin-B	UBB	229
50	P08670	VIME_HUMAN	Vimentin	VIM	466

B.

	Accession		Protein name	Gene Name	Sequence length (a.a.)
**1**	P60709	ACTB_HUMAN	Actin, cytoplasmic 1 (Beta-actin)	ACTB	375
**2**	P68032	ACTC_HUMAN	Actin, alpha cardiac muscle 1 (Alpha-cardiac actin)	ACTC1	377
**3**	Q09666	AHNK_HUMAN	Neuroblast differentiation-associated protein AHNAK (Desmoyokin)	AHNAK	5890
**4**	P02768	ALBU_HUMAN	Serum albumin	ALB GIG20	609
**5**	P04083	ANXA1_HUMAN	Annexin A1 (Annexin I) (Annexin-1) (Calpactin II)	ANXA1 ANX1 LPC1	346
**6**	P07355	ANXA2_HUMAN	Annexin A2 (Annexin II) (Annexin-2)	ANXA2	339
**7**	P09525	ANXA4_HUMAN	Annexin A4 (35-beta calcimedin) (Annexin IV) (Annexin-4)	ANXA4	319
**8**	Q13938	CAYP1_HUMAN	Calcyphosin (Calcyphosine)	CAPS	275
**9**	P12111	CO6A3_HUMAN	Collagen alpha-3(VI) chain	COL6A3	3177
**10**	P15924	DESP_HUMAN	Desmoplakin (DP)	DSP	2871
**11**	P68104	EF1A1_HUMAN	Elongation factor 1-alpha 1 (EF-1-alpha-1)	EEF1A1	462
**12**	P35555	FBN1_HUMAN	Fibrillin-1	FBN1	2871
**13**	P21333	FLNA_HUMAN	Filamin-A (FLN-A)	FLNA	2647
**14**	P02751	FINC_HUMAN	Fibronectin (FN)	FN1	2386
**15**	P69905	HBA_HUMAN	Hemoglobin subunit alpha (Alpha-globin)	HBA1	142
**16**	P68871	HBB_HUMAN	Hemoglobin subunit beta (Beta-globin)	HBB	147
**17**	P62805	H4_HUMAN	Histone H4	HIST1H4A	103
**18**	P22626	ROA2_HUMAN	Heterogeneous nuclear ribonucleoproteins A2/B1 (hnRNP A2/B1)	HNRNPA2B1	353
**19**	Q86YZ3	HORN_HUMAN	Hornerin	HRNR	2850
**20**	P07900	HS90A_HUMAN	Heat shock protein HSP 90-alpha) (HSP 86) (HSP86)	HSP90AA1	732
**21**	P0DMV8	HS71A_HUMAN	Heat shock 70 kDa protein 1A (HSP70-1)	HSPA1A	641
**22**	P11021	BIP_HUMAN	Endoplasmic reticulum chaperone BiP (EC 3.6.4.10)	HSPA5	654
**23**	P98160	PGBM_HUMAN	Basement membrane-specific heparan sulfate proteoglycan core protein (HSPG)	HSPG2	4391
**24**	P04264	K2C1_HUMAN	Keratin, type II cytoskeletal 1 (CK-1)	KRT1	644
**25**	P13645	K1C10_HUMAN	Keratin, type I cytoskeletal 10 (CK-10)	KRT10	584
**26**	P13646	K1C13_HUMAN	Keratin, type I cytoskeletal 13 (CK-13)	KRT13	458
**27**	P02533	K1C14_HUMAN	Keratin, type I cytoskeletal 14 (CK-14)	KRT14	472
**28**	P08779	K1C16_HUMAN	Keratin, type I cytoskeletal 16 (CK-16)	KRT16	473
**29**	Q04695	K1C17_HUMAN	Keratin, type I cytoskeletal 17 (CK-17)	KRT17	432
**30**	P08727	K1C19_HUMAN	Keratin, type I cytoskeletal 19 (CK-19)	KRT19	400
**31**	P35908	K22E_HUMAN	Keratin, type II cytoskeletal 2 epidermal (CK-2e)	KRT2	639
**32**	P13647	K2C5_HUMAN	Keratin, type II cytoskeletal 5 (CK-5)	KRT5	590
**33**	P02538	K2C6A_HUMAN	Keratin, type II cytoskeletal 6A (CK-6A)	KRT6A	564
**34**	P04259	K2C6B_HUMAN	Keratin, type II cytoskeletal 6B (CK-6B)	KRT6B	564
**35**	P48668	K2C6C_HUMAN	Keratin, type II cytoskeletal 6C (CK-6C)	KRT6C	564
**36**	P08729	K2C7_HUMAN	Keratin, type II cytoskeletal 7 (CK-7)	KRT7	469
**37**	P05787	K2C8_HUMAN	Keratin, type II cytoskeletal 8 (CK-8)	KRT8	483
**38**	P35527	K1C9_HUMAN	Keratin, type I cytoskeletal 9 (CK-9)	KRT9	623
**39**	P02545	LMNA_HUMAN	Prelamin-A/C [Cleaved into: Lamin-A/C	LMNA	664
**40**	P35579	MYH9_HUMAN	Myosin-9 (Cellular myosin heavy chain, type A)	MYH9	1960
**41**	P14618	KPYM_HUMAN	Pyruvate kinase PKM (EC 2.7.1.40) (CTHBP)	PKM	531
**42**	Q15149	PLEC_HUMAN	Plectin (PCN) (PLTN)	PLEC	4684
**43**	Q06830	PRDX1_HUMAN	Peroxiredoxin-1 (NKEF-A)	PRDX1	199
**44**	Q01082	SPTB2_HUMAN	Spectrin beta chain, non-erythrocytic 1 (Beta-II spectrin)	SPTBN1	2364
**45**	P02787	TRFE_HUMAN	Serotransferrin (Transferrin)	TF	698
**46**	P60174	TPIS_HUMAN	Triosephosphate isomerase (TIM)	TPI1	286
**47**	Q71U36	TBA1A_HUMAN	Tubulin alpha-1A chain (Alpha-tubulin 3)	TUBA1A	451
**48**	P68371	TBB4B_HUMAN	Tubulin beta-4B chain (Tubulin beta-2 chain)	TUBB4B	445
**49**	P0CG47	UBB_HUMAN	Polyubiquitin-B	UBB	229
**50**	P08670	VIME_HUMAN	Vimentin	VIM	466

C.

	Accession		Protein name	Gene Name	Sequence length (a.a.)
**1**	P60709	ACTB_HUMAN	Actin, cytoplasmic 1 (Beta-actin)	ACTB	375
**2**	P68032	ACTC_HUMAN	Actin, alpha cardiac muscle 1 (Alpha-cardiac actin)	ACTC1	377
**3**	P02768	ALBU_HUMAN	Serum albumin	ALB	609
**4**	P07355	ANXA2_HUMAN	Annexin A2 (Annexin II) (Annexin-2)	ANXA2	339
**5**	Q13938	CAYP1_HUMAN	Calcyphosin (Calcyphosine)	CAPS	275
**6**	P59665	DEF1_HUMAN	Neutrophil defensin 1	DEFA1	94
**7**	Q02413	DSG1_HUMAN	Desmoglein-1	DSG1	1049
**8**	P15924	DESP_HUMAN	Desmoplakin (DP)	DSP	2871
**9**	P68104	EF1A1_HUMAN	Elongation factor 1-alpha 1 (EF-1-alpha-1)	EEF1A1	462
**10**	P06733	ENOA_HUMAN	Alpha-enolase	ENO1	434
**11**	P04406	G3P_HUMAN	Glyceraldehyde-3-phosphate dehydrogenase	GAPDH	335
**12**	P69905	HBA_HUMAN	Hemoglobin subunit alpha (Alpha-globin)	HBA1	142
**13**	P69892	HBG2_HUMAN	Hemoglobin subunit gamma-2	HBG2	147
**14**	Q93077	H2A1C_HUMAN	Histone H2A type 1-C	HIST1H2AC	130
**15**	O60814	H2B1K_HUMAN	Histone H2B type 1-K	HIST1H2BK	126
**16**	P62805	H4_HUMAN	Histone H4	HIST1H4A	103
**17**	P22626	ROA2_HUMAN	Heterogeneous nuclear ribonucleoproteins A2/B1 (hnRNP A2/B1)	HNRNPA2B1	353
**18**	P61978	HNRPK_HUMAN	Heterogeneous nuclear ribonucleoprotein K	HNRNPK	463
**19**	Q86YZ3	HORN_HUMAN	Hornerin	HRNR	2850
**20**	P07900	HS90A_HUMAN	Heat shock protein HSP 90-alpha) (HSP 86) (HSP86)	HSP90AA1	732
**21**	P08238	HS90B_HUMAN	Heat shock protein HSP 90-beta	HSP90AB1	724
**22**	P11021	BIP_HUMAN	Endoplasmic reticulum chaperone BiP (EC 3.6.4.10)	HSPA5	654
**23**	P14923	PLAK_HUMAN	Junction plakoglobin	JUP	745
**24**	Q5T749	KPRP_HUMAN	Keratinocyte proline-rich protein	KPRP	579
**25**	P04264	K2C1_HUMAN	Keratin, type II cytoskeletal 1 (CK-1)	KRT1	644
**26**	P13645	K1C10_HUMAN	Keratin, type I cytoskeletal 10 (CK-10)	KRT10	584
**27**	P13646	K1C13_HUMAN	Keratin, type I cytoskeletal 13 (CK-13)	KRT13	458
**28**	P02533	K1C14_HUMAN	Keratin, type I cytoskeletal 14 (CK-14)	KRT14	472
**29**	P08779	K1C16_HUMAN	Keratin, type I cytoskeletal 16 (CK-16)	KRT16	473
**30**	Q04695	K1C17_HUMAN	Keratin, type I cytoskeletal 17 (CK-17)	KRT17	432
**31**	P08727	K1C19_HUMAN	Keratin, type I cytoskeletal 19 (CK-19)	KRT19	400
**32**	P35908	K22E_HUMAN	Keratin, type II cytoskeletal 2 epidermal (CK-2e)	KRT2	639
**33**	Q15323	K1H1_HUMAN	Keratin, type I cuticular Ha1	KRT31	416
**34**	P19013	K2C4_HUMAN	Keratin, type II cytoskeletal 4	KRT4	534
**35**	P13647	K2C5_HUMAN	Keratin, type II cytoskeletal 5 (CK-5)	KRT5	590
**36**	P02538	K2C6A_HUMAN	Keratin, type II cytoskeletal 6A (CK-6A)	KRT6A	564
**37**	P04259	K2C6B_HUMAN	Keratin, type II cytoskeletal 6B (CK-6B)	KRT6B	564
**38**	P48668	K2C6C_HUMAN	Keratin, type II cytoskeletal 6C (CK-6C)	KRT6C	564
**39**	Q01546	K22O_HUMAN	Keratin, type II cytoskeletal 2 oral	KRT76	638
**40**	Q7Z794	K2C1B_HUMAN	Keratin, type II cytoskeletal 1b	KRT77	578
**41**	Q8N1N4	K2C78_HUMAN	Keratin, type II cytoskeletal 78	KRT78	520
**42**	P05787	K2C8_HUMAN	Keratin, type II cytoskeletal 8 (CK-8)	KRT8	483
**43**	P78386	KRT85_HUMAN	Keratin, type II cuticular Hb5	KRT85	507
**44**	O43790	KRT86_HUMAN	Keratin, type II cuticular Hb6	KRT86	486
**45**	P35527	K1C9_HUMAN	Keratin, type I cytoskeletal 9 (CK-9)	KRT9	623
**46**	Q71U36	TBA1A_HUMAN	Tubulin alpha-1A chain (Alpha-tubulin 3)	TUBA1A	451
**47**	P07437	TBB5_HUMAN	Tubulin beta chain (Tubulin beta-5 chain)	TUBB	444
**48**	P68371	TBB4B_HUMAN	Tubulin beta-4B chain (Tubulin beta-2 chain)	TUBB4B	445
**49**	P0CG47	UBB_HUMAN	Polyubiquitin-B	UBB	229
**50**	P08670	VIME_HUMAN	Vimentin	VIM	466
