# Supplementary material for: New insights on congenital pulmonary airways malformations revealed by proteomic analyses
Source: Orphanet J Rare Dis. 2019 Nov 28;14:272. doi: 10.1186/s13023-019-1192-4 (PMC6883702; doi:10.1186/s13023-019-1192-4)
Supplement: Supplementary file 3 — Additional file 3: Figure S3. List of protein candidates. A. List of proteins significantly upregulated in CPAM 1 and fetal bronchi, as compared to CPAM 2 and fetal canaliculi determined with the GO tool from PANTHER (GO consortium, PANTHER, maintained by Thomas laboratory, University of Southern California). B. List of proteins significantly upregulated in CPAM 2 and the possible pathways in which they are involved, determined with the GO tool from PANTHER (GO consortium, PANTHER, maintained by Thomas laboratory, University of Southern California). [file 13023_2019_1192_MOESM3_ESM.pptx]

## Slide 1
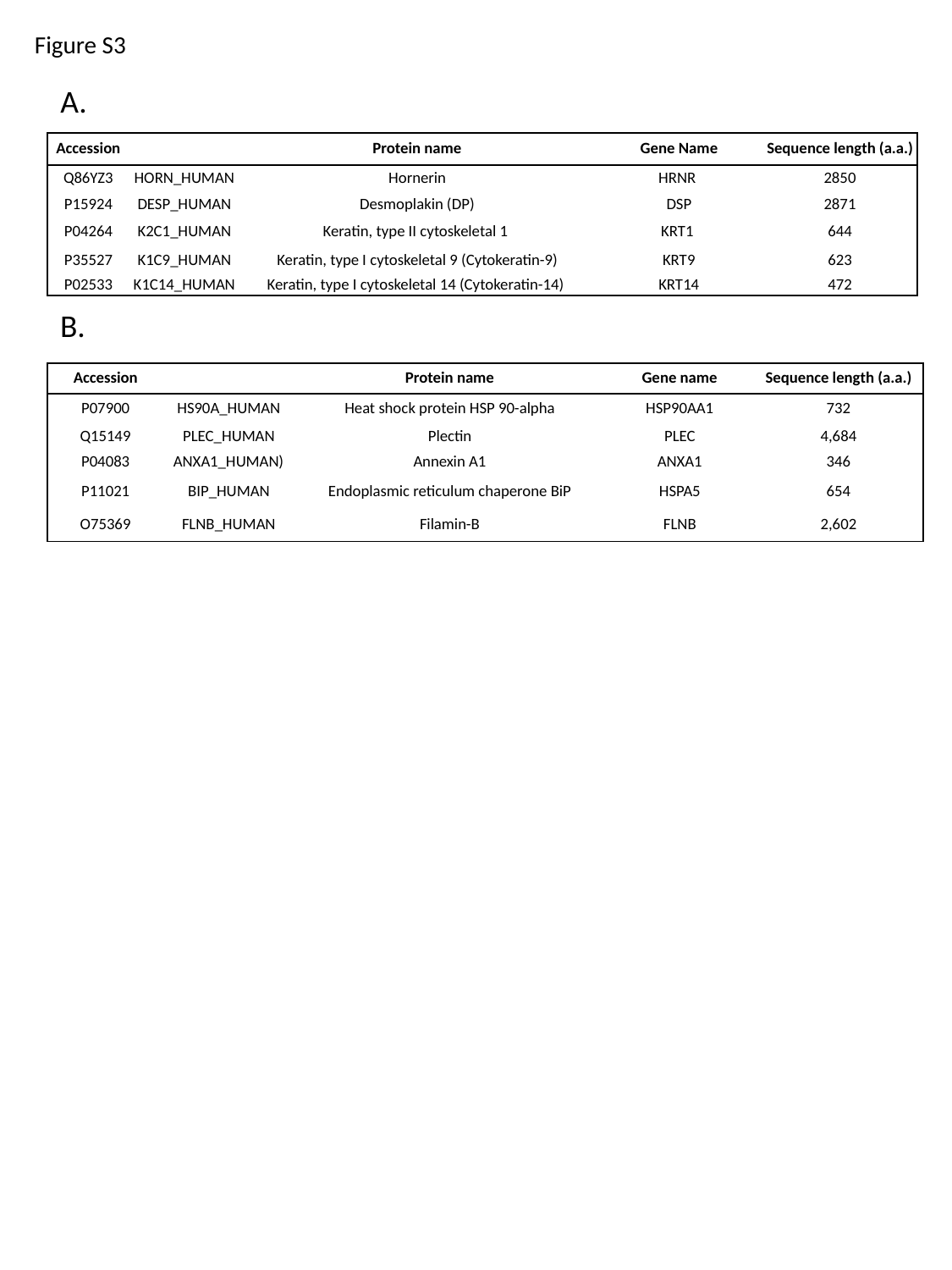

Figure S3
A.
| Accession | | Protein name | Gene Name | Sequence length (a.a.) |
| --- | --- | --- | --- | --- |
| Q86YZ3 | HORN\_HUMAN | Hornerin | HRNR | 2850 |
| P15924 | DESP\_HUMAN | Desmoplakin (DP) | DSP | 2871 |
| P04264 | K2C1\_HUMAN | Keratin, type II cytoskeletal 1 | KRT1 | 644 |
| P35527 | K1C9\_HUMAN | Keratin, type I cytoskeletal 9 (Cytokeratin-9) | KRT9 | 623 |
| P02533 | K1C14\_HUMAN | Keratin, type I cytoskeletal 14 (Cytokeratin-14) | KRT14 | 472 |
B.
| Accession | | Protein name | Gene name | Sequence length (a.a.) |
| --- | --- | --- | --- | --- |
| P07900 | HS90A\_HUMAN | Heat shock protein HSP 90-alpha | HSP90AA1 | 732 |
| Q15149 | PLEC\_HUMAN | Plectin | PLEC | 4,684 |
| P04083 | ANXA1\_HUMAN) | Annexin A1 | ANXA1 | 346 |
| P11021 | BIP\_HUMAN | Endoplasmic reticulum chaperone BiP | HSPA5 | 654 |
| O75369 | FLNB\_HUMAN | Filamin-B | FLNB | 2,602 |
