# Supplementary material for: New insights on congenital pulmonary airways malformations revealed by proteomic analyses
Source: Orphanet J Rare Dis. 2019 Nov 28;14:272. doi: 10.1186/s13023-019-1192-4 (PMC6883702; doi:10.1186/s13023-019-1192-4)
Supplement: Supplementary file 4 — Additional file 4. Supplementary material and methods. [file 13023_2019_1192_MOESM4_ESM.docx]

**Supplementary material and methods**

**CPAM description**

CPAM classification mostly relies on the modified Stocker classification (^13 15 16^) that recognizes 5 CPAM types, the most frequent being type 1 (large cysts) and 2 (small cysts). Type 4 is considered as pleuropulmonary blastoma, therefore representing a malignant tumor instead of a malformation. Lesions were classified according mainly to their histological features, gross findings being also recorded. To qualify for the diagnosis of CPAM (instead of intrapulmonary bronchogenic cyst), the cysts have to merge at least focally with the surrounding alveoli. CPAM types are determined according to the following histopathological criteria (^13^). Type 1 CPAM is composed of cysts mostly lined by ciliated pseudostratified columnar epithelium, with occasional mucus producing cells. The thick cyst wall contains prominent smooth muscle and elastic tissue, and rarely an island of cartilage. Type 2 CPAM is composed of more uniform and smaller, multiple evenly spaced cysts, lined by ciliated cuboidal to columnar epithelium, with only rare pseudostratification. Mucous cells and cartilage are not seen, except when belonging to entrapped normal bronchi. CPAM 2 cyst wall is thin, composed of loose connective tissue and discontinuous bands of smooth muscle and elastic tissue. Sequestration was defined by the presence of systemic blood supply and gross identification and histological confirmation of the presence of a thick-walled elastic and/or muscular artery intermingled.

Diagnosis was confirmed by thoracic CT-scan performed at the age of 6 months and surgery was performed between 6 and 18 months of age. Surgical samples were routinely processed for histological analysis. Gross examination was performed prior to fixation in formaldehyde 4%; no tissue inflation was performed. After fixation, all cystic lesions were entirely embedded in paraffin together with control lung tissue, whenever feasible. Each analysis was performed in 3 to 7 different patients of CPAM 1, CPAM 2 and on their healthy adjacent lung. For human fetal samples, the only information provided was gestational age and whether there were any known genetic or structural abnormalities. Fetal lung samples of 16 GW, corresponding to the time of diagnosis, were analyzed.

**Immunohistochemistry and staining quantification**

Slides were prepared as previously described (^38^). Slides were incubated with the different antibodies: 1) rabbit polyclonal anti- SOX2 1:1000 (Seven Hills Bioreagents, WRAB-1236), 2) rabbit polyclonal anti-SOX9 1:1000 (Merck, AB5535), 3) rabbit polyclonal anti-Pro-SPC 1:500 (Merck, AB3786), 4) mouse monoclonal anti-ACTA2 1:250 (kind gift of Dr. ML Bochaton Piallat ^39^), 5) mouse monoclonal anti-Krt17 1:250 (Santa Cruz, sc-393002), 6) mouse monoclonal anti-MUC5AC 1:250 (Santa-Cruz, sc-33667), 6) mouse monoclonal anti-Ki67 1:1000 (Agilent, M7240). Different IgG were used as specificity control for IHC: 1) Normal rabbit IgG 5µg/ml (Invitrogen, 10500C), 2) mouse IgG1 0.5 µg/ml (DAKO, X0931) 3) mouse IgG2a 0.1 µg/ml (DAKO, X0943). 3,3’ – diaminobenzidine (DAB) substrate (DAKO) or UltraView RED detection kit were used for antibodies detection (Roche, Ventana Medical Systems Inc., Arizona, USA). Slides were counterstained with Mayer’s Hemalun. Finally, glass covers were applied on slides with mounting medium. Quantitative immunohistochemistry was performed as previously described (^40^). Slides for morphometric analysis were scanned at 20x magnification and high resolution using a fully automated Axio Scan.Z1 equipped with a Plan-Apochromat 20x/0.8 objective M27 (Carl Zeiss, Jena, Germany). Immunohistochemistry staining was quantitatively analyzed using the image-processing program ImageJ v1.49 software (NIH, <http://rsb.info.nih.gov/ij/>). Cross-sectional area of the different epithelium was manually drawn in order to cover the whole epithelial surface. Subsequently, positive cells within the cross-sectional area were automatically discriminated from the unstained portions of the specimens according to hue (i.e. dominant colour tone), brightness (i.e. colour intensity) and saturation (i.e. colour purity) components, and quantified. Results were calculated as area of immunostaining/ epithelium area. Images for illustrations were taken by means of Axio Scan.Z1 equipped with a Plan-Apochromat 20x/0.8 objective M27.

**Proteomic analysis and LMD**

For total sample analysis, 10μm paraffin slices were collected in microcentrifuge tubes (Eppendorf, Hambourg, Germany). the qProteome FFPE-tissue kit (Qiagen, Hilden, Germany), following the manufacturer instructions. Protein extraction was performed using RapiGest buffer (Waters Corporation, Massachussets, USA) for microlaser dissection experiments. Briefly, for each condition, 3 sections of 10 μm of tissue were collected in an eppendorf tube, followed by two consecutive washes in Xylene for 10 minutes each. The slides were then rehydrated in ethanol baths. After the rehydration, tissue pellets were air-dryed and 100 μl of lysis buffer (RapiGest 0.2% in 40 Mm Tris-HCl, 5Mm DTT) was added. The samples were subsequently sonicated, then incubated at 98° for 20 min and at 80° for 2 hours in a bench heating-block.

For LMD analysis, paraffin-embedded sections (thickness: 10 μm) were mounted on PET-membrane frame slides (PET-Membrane, No.11505151, Microdissect GMBH, Herborn, Germany). Two sections per sample were used to pool a larger number of cells for each condition. The slides were deparaffinized and rehydrated in ethanol baths. After the rehydration, tissue pellets were air-dryed and 100 μl of lysis buffer (RapiGest 0.2% in 40 Mm Tris-HCl, 5Mm DTT) was added. The samples were subsequently sonicated for 5 cycles and incubated for 5 minutes at 98°C and 15 minutes at 80°C. Samples were cooled on ice for 5 minutes then stored at -20°C until further analysis.

Since protein concentration for this quantity of tissue was poorly accurate, we used previously published methods to estimate the corresponding cell numbers (28–30). Approximately 15 areas, posteriorly-assessed as ranging from 160000 - 300000 μm2, corresponding roughly to 1500 epithelial cells were cut and captured by gravity in microtubes (Axygen Scientific Inc, California, USA), using the widefield Upright Leica DM 6500 microscope (LEICA, Wetzlar, Germany) coupled with Cryslas laser (Wavelength: 355 nm). To attest the reproducibility of the proteomic extraction and analysis by LC/MS-MS, we sequenced six LMD-derived samples across two different experiments. We found that the highest variation between two replicates from the same biological sample was 55.7%, whereas the smaller variation was 22.8%, showing that there is a reasonable variability arising from experimental manipulation (^41 42^).
